# Correction: Measuring Mosquito-borne Viral Suitability in Myanmar and Implications for Local Zika Virus Transmission

**DOI:** 10.1371/currents.outbreaks.9934c8779f27f8fa6e4d59d3197dff85

**Published:** 2018-10-10

**Authors:** - PLOS Currents

## Correction

The authors are listed out of order. Please view the correct author order, affiliations, and citation here:

Pablo Noel Perez-Guzman^1,2^, Uri Obolski^3^, Luiz Carlos Junior Alcantara^4^, Maricelia M. de Lima^4^, Elizabeth A. Ashley^5,6^, Frank Smithuis^5,6,7^, Peter Horby^7^, Richard J. Maude^6,7,8,9^, Zaw Lin^10^, Aye Mon Mon Kyaw^10^, José Lourenço^3^

1 Department of Global Health and Tropical Medicine, University of Oxford, UK; 2 Department of Infectious Disease Epidemiology, Imperial College, London, UK; 3 Department of Zoology, University of Oxford, UK; 4 Laboratory of Haematology, Genetics and Computational Biology, FIOCRUZ, Brazil; 5 Myanmar-Oxford Clinical Research Unit, Yangon; 6 Centre for Tropical Medicine and Global Health, Nuffield Department of Medicine, University of Oxford, UK; 7 Nuffield Department of Medicine, University of Oxford, UK; 8 Mahidol-Oxford Tropical Medicine Research Unit, Faculty of Tropical Medicine, Mahidol University,Thailand; 9 Harvard TH Chan School of Public Health, Harvard University, Boston, USA; 10 Myanmar Ministry of Health and Sports, Naypyidaw, Myanmar

Perez-Guzman PN, Obolski U, Carlos Junior Alcantara L, de Lima MM, Ashley EA, Smithuis F, Horby P, Maude RJ, Lin Z, Kyaw AMM, Lourenço J. Measuring Mosquito-borne Viral Suitability in Myanmar and Implications for Local Zika Virus Transmission. PLOS Currents Outbreaks. 2018 Sep 28 . Edition 1. doi: 10.1371/currents.outbreaks.7a6c64436a3085ebba37e5329ba169e6.
